# From semi-starvation to the stage: a case report on indicators of low energy availability in a drug-free bodybuilder during contest preparation and peak week

**DOI:** 10.3389/fnut.2024.1465001

**Published:** 2024-11-13

**Authors:** Alex J. Ritson, Lyle McDonald, Joseph Agu, Laurent G. Bannock

**Affiliations:** ^1^Department of Education, Institute of Performance Nutrition, London, United Kingdom; ^2^Bodyrecomposition Ltd., Austin, TX, United States; ^3^School of Medicine, University of Nottingham, Nottingham, United Kingdom

**Keywords:** natural bodybuilding, relative energy deficiency in sport, endocrine function, IOC REDs CAT2, case report, total testosterone, free triiodothyronine

## Abstract

Natural bodybuilding competitions involve periods of low energy availability (EA) combined with resistance training and high-protein diets to achieve extreme leanness. This study tracked a drug-free bodybuilder adopting evidence-based nutrition practices during 18 weeks of contest preparation. We measured endocrine function, resting energy expenditure, respiratory exchange ratio, body composition, resting heart rate, oral temperature, mood, and strength performance. Endocrine function was remeasured after 2 days of energy repletion. From baseline to week 18, free triiodothyronine (T3) and total testosterone (TT) fell into clinically low (2.7 pmol/L^−1^) and sub-clinically low (9.1 nmol/L^−1^) ranges. Resting energy expenditure decreased by −519 kcal (REE_ratio_ 0.78), and respiratory exchange ratio decreased from 0.95 to 0.85. Body mass reduced by −5.1 kg, with a sum of eight skinfold loss of −15.7 mm. Correlations were observed between body mass and decreases in oral temperature (r = 0.674, *p* = 0.002) and resting heart rate (r = 0.560, *p* = 0.016). Mood remained stable until the final 2 weeks and relative one-repetition maximum decreased in the squat (−5.4%), bench (−2.6%), and deadlift (−3.6%). Following 2 days of modest energy repletion, free T3 increased (18.5%), returning to sub-clinically low values (3.2 pmol/L^−1^), whereas TT fell (−20.9%), reaching clinically low values (7.2 nmol/L^−1^). These results offer insight into the dynamics of T3 and TT following a short-term period of modest energy repletion and further information on indicators of low EA during chronic energy restriction.

## 1 Introduction

Natural bodybuilding is a sport that blends physical skill with artistic presentation (1). Bodybuilding competitions are judged on an athlete’s muscular size, symmetry, definition, and posing skills (2). To obtain the desired muscle definition, athletes undergo a period of calorie restriction and increased activity energy expenditure to lose fat mass, coupled with nutrition and resistance training practices to preserve skeletal muscle (3). After an extended period of energy restriction, athletes often increase their calorie, carbohydrate, and sodium intake several days before competition to increase muscle glycogen stores and intracellular fluid to accentuate muscle volume, known as “peaking” (4). Collectively, these strategies encompass a bodybuilder’s contest preparation (“contest prep”).

Natural bodybuilding is unique in that athletes willingly subject themselves to semi-starvation to achieve their contest condition, cognizant of the disruptions to physiological and psychological function, which are largely “adaptable” states of low energy availability (EA; transient, reversible signs of body system suppression with minimal long-term adverse health effects) (5, 6), but can manifest into “problematic” low EA (signs of body system suppression with long-term adverse health effects) in some bodybuilders (7) and other physique competitors (8).

Since the first prospective studies associated low EA with reduced triiodothyronine (T3) and luteinizing hormone (LH) dysregulation in healthy, sedentary women three decades ago (9, 10), mirroring observations seen in amenorrhoeic female athletes (11, 12), a growing body of research has surfaced on indicators of low EA in athletes. Consolidating low EA research with athlete testing, validation, and usability, the IOC REDs Clinical Assessment Tool Version 2 (IOC REDs CAT2) was developed to detect signs of problematic low EA in athletes (13).

Two primary indicators of low EA in Step 2 of the IOC REDs CAT2 for males are clinically low and sub-clinically low (within the lowest 25% quartile of the reference range) total or free T3 and testosterone (14). During chronic states of energy restriction in natural bodybuilders, T3 and testosterone levels reach clinically low values (5, 6); however, both can recover to within normal physiological ranges and baseline values in 1–3 months following an increase in energy intake and body mass (BM) post contest prep (5, 6). Experimental research in a male combat athlete and soldiers following a chronic state of energy restriction has shown that energy repletion that far exceeds total daily energy expenditure rates restores T3 and testosterone levels to within normal physiological ranges and baseline values within several days to a week, albeit with pronounced increases in BM (15, 16).

Although the diagnostic measures of T3 and testosterone serve as primary indicators of problematic low EA in male athletes, the measurements themselves adapt quickly to changes in energy intake and EA. While substantial energy repletion intakes and rapid increases in BM are practiced by combat and physique athletes following the termination of a phase of energy restriction (16–18) they do not reflect the acute energy repletion (“refeed”) practices of physique athletes during continued energy restriction (19, 20) nor the modest fluctuations in energy balance and EA of other athletes between training (and matches) and rest days (21–24).

A lack of research exists on the transient nature of T3 and testosterone levels after short-term, modest energy repletion following a period of energy restriction. Understanding how T3 and testosterone levels respond to acute changes in energy intake following a phase of energy restriction and low EA would clarify their responsiveness and assist physicians in accurately timing and interpreting these diagnostic measurements of problematic low EA in certain athletes. In addition, such insights would help discern whether the dietary practice of refeeding (2 days) in bodybuilding is efficacious for attenuating endocrine adaptations during ongoing energy restriction (25).

Reductions in T3 have been shown to coincide with stepwise suppressions in REE and menstrual disturbance (26), the latter being a primary marker of low EA in women (27). The assessment of low EA using REE is commonly depicted as a REE_ratio_ of measured REE (mREE) via indirect calorimetry to a predictive estimate of REE (pREE) with a cut-off ratio of <0.90 (mREE/pREE) (27). However, REE remains a “potential” marker in the IOC REDs CAT2 assessment model due to a lack of consistency in the literature (13). This discrepancy may be partly due to predictive equations for determining the REE_ratio_ being non-specific to the population studied, exercise being performed close to measurement, and the lack of weight stabilisation before an intervention (27).

Therefore, this case study serves as a lens to assess the effects of two primary indicators of low EA in a male bodybuilder during chronic energy restriction and 2 days of modest energy repletion in the athlete’s peaking phase. It also provides further information on REE and REE_ratio_ during chronic energy restriction using an athlete-specific predictive equation and adopting recommended pre-assessment procedures. Lastly, we offer insight into field-based measurements accessible to the research-active practitioner on aspects of physiology, psychology, and strength performance during a chronic phase of energy restriction. Collectively, our findings offer further empirical evidence to contribute to the shortage of research on low EA in male athletes and assist in contextualizing low EA markers using tools such as the IOC REDs CAT2.

## 2 Case description and assessment methods

### 2.1 Case presentation

The athlete (25 years, BMI = 26.2 kg.m^2^) was a drug-free Caucasian male amateur bodybuilder competing in his fourth natural bodybuilding competition in the Natural Physique Association. The athlete adhered to a varied, flexible diet with no food restrictions. As a qualified Personal Trainer and competitive powerlifter, the athlete was aware of the biases, typical errors and standardization requirements with tracking their dietary intake and BM and had ample experience after maintaining a detailed dietary/BM record during previous contest preps and powerlifting competitions. The athlete tracked their dietary intake using MyFitnessPal (MyFitnessPal Inc. CA, USA) and BM daily throughout the 18-week intervention. The athlete was experienced with testing their one-repetition maximum (1RM) on the three multi-joint exercises: back squat, bench press, and deadlift. The athlete was not taking any prescribed medication. He was a non-smoker and supplemented with creatine monohydrate, omega 3 (fish oil), vitamin D3, whey protein, casein, citrulline malate, and beta-alanine.

### 2.2 Metabolic assessment

**Figure 1:**
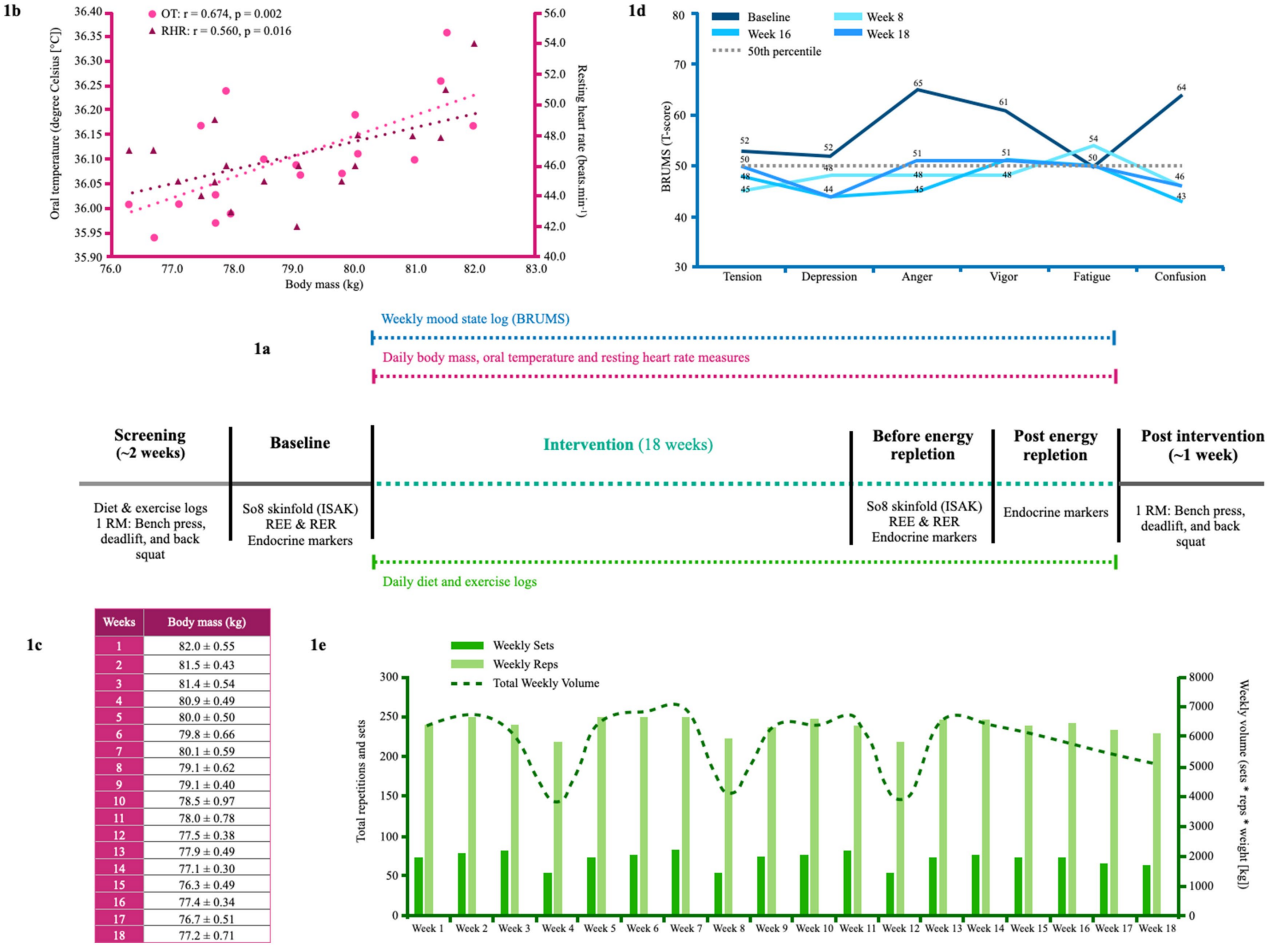
**(a)** The intervention timeline. **(b)** The relationship between oral temperature, RHR and body mass from week 1 to week 18. **(c)** Weekly body mass averages in kilograms taken at the athlete's home. **(d)** BRUMS scale at baseline, week 8, week 16, and week 18. The horizontal dashed line represents the normative mean. **(e)** Overview of weekly resistance volume and weekly sets and reps completed from week 1 to week 18. 1RM, one repetition maximum; BRUMS, Brunel mood scale; OT, oral temperature; REE, resting energy expenditure; RER, respiratory exchange rate; RHR; resting heart rate; So8, sum of eight.

An overview of assessments is shown in the intervention timeline in Figure 1a. Resting energy expenditure (REE) and respiratory exchange ratio (RER) were determined using an online gas analysis system (MetaLyzer 3B, Cortex) at baseline and beginning of week 18 (Table 1). The gas analyzer was calibrated before testing, and environmental conditions during testing were 23.8 ± 1.3 degrees Celsius. For the REE and RER assessments, the athlete was required to lie still on a bed in a supine position for 30 min before the assessment. There was no visual or auditory stimulation throughout the measurement period. The athlete was requested to abstain from strenuous physical activity the day before or the morning of the test. For the initial assessment, the athlete maintained a stable BM (≤1% deviation) for 2 weeks before the assessment. The athlete’s REE_ratio_ was calculated as the division of measured REE (mREE) and predictive REE (pREE). We chose a validated prediction equation that best aligned with the physical characteristics and sport of the athlete, specifically, the bodyweight-based predictive equation derived from physique athletes (28). Although the equation was determined in a mixed cohort of users and non-users of anabolic-androgenic steroids, the sub-analysis of self-reported non-users (*n* = 20) showed a strong positive agreement (r = 0.93; 29 kcal.d) with the equation.

**Table 1:** Anthropometric and metabolic measurements at baseline and week 18.

Athlete characteristics	Baseline	Week 18	Absolute change (%)
Body mass (kg)	81.8	76.7	−5.1 (−6.2%)
BMI (kg.m^2^)	26.2	24.6	−1.6 (−6.1%)
Waist/hip ratio	0.84	0.79	−0.05 (−6.0%)
ISAK sum of 8 skinfolds (mm)	50.8 (z-score: −2.3)	35.1 (z-score: −2.6)	−15.7 (−30.9%)
REE (measured; kcal.d^−1^)	2015	1,496	−519 (−25.8%)
REE (predicted; kcal.d^−1^)	2039	1912	−127 (−6.2%)
mREE/pREE ratio	0.99	0.78	−0.21 (−21.2%)
RER	0.95	0.85	−0.10 (−10.5%)

All data presented are measurements taken at baseline (before the intervention) and at the beginning of week 18 (before the peaking phase). The sum of skinfolds is the total of eight skinfold sites (tricep, subscapular, bicep, iliac crest, supraspinale, abdominal, front thigh [straight leg], and medial calf) taken in accordance with the standards of the International Society for the Advancement of Kinanthropometry (ISAK). All skinfold measurements were performed directly after the athlete’s REE and RER measurements. REE, resting energy expenditure; RER, respiratory exchange ratio; m, measured; p, predicted.

### 2.3 Anthropometric assessment

The athlete underwent two body composition assessments at baseline and at the beginning of week 18. Height (Stadiometer, Seca, UK), BM (Seca, UK), skinfolds (Harpenden® calipers) and girth measurements (Lufkin® steel tape) were performed by two certified anthropometrist’s (Level 1) by the International Society for the Advancement of Kinanthropometry (ISAK) using the same calibrated equipment. Level 1 ISAK anthropometrists’ must not exceed a technical error of measurement (TEM) of 7.5% for any skinfold nor 1.5% for any other measure to obtain their certification. Skinfold and girth measurements were performed in duplicate or triplicate, depending on whether the difference of the first and second measurements exceeded 5% (skinfold) and 1% (all other measurements), following which a mean value was obtained (results shown in Table 1). Due to large discrepancies in body fat predictive equations when using skinfold thickness measurements (29), the athlete’s values were presented as a sum of eight sites; this also prevented the study from directly quantifying the athlete’s EA. Throughout the intervention, the athlete tracked his weight daily in minimal clothing to the nearest tenth of a pound (converted to kilograms in this manuscript) immediately upon waking using a standard calibrated electrical scale (Tanita HD 386 Super Compact Digital Scale) (Figure 1c).

### 2.4 Blood parameters

Endocrine markers (Table 2) were obtained at baseline, beginning of week 18 and 2 days following energy repletion. Measurements were taken by venipuncture of the antecubital vein of the left arm at 0900-0920 following a 12-h overnight fast at each time point. Endocrine markers were conducted using the electrochemiluminescence immunoassay (ECLIA) method on the cobas e 602 analyzer (Roche Diagnostics). Measurements were performed by The Doctors Laboratory (TDL, London, UK). The inter assay coefficient of variation (CoV) and sensitivity (lowest detection limit) for all endocrine markers were sourced from Roche Diagnostics validation reports, as replicate measurements were not performed internally; thus, precision was determined based on the manufacturer’s reported CoV% for similar mean concentrations (see Supplementary Table 1).

**Table 2:** Endocrine markers at baseline, week 18 and 2 days of energy repletion (peaking).

Endocrine markers	Baseline	Beginning of week 18	Post 2 days of energy repletion
Follicular stimulating hormone (IU/L^−1^) Ref values: 1.5–12.4	6.7	6.0	5.7
Luteinizing hormone (IU/L^−1^) Ref values: 1.7–8.6	3.8	3.6	2.9
DHEA sulphate (μmol/L^−1^) Ref values: 4.34–12.2	10.3	9.3	9.7
Total testosterone (nmol/L^−1^) Ref values: 8.6–29.0 Lowest 25% quartile of the ref values: 8.6–13.7 nmol/L	14.4	9.1^*^	7.2^**^
SHBG (nmol/L^−1^) Ref values: 18.3–54.1	27	28	26
Testosterone/SHBG ratio Ref values: 24–104	53.3	32.5	27.7
Cortisol (nmol/L^−1^) Ref values: 166–507 Morning 6–10 am	482	449	454
Free triiodothyronine (pmol/L^−1^) Ref values: 3.1–6.8 Lowest 25% quartile of the ref values: 3.1–4.0 pmol/L	4.3	2.7^**^	3.2^*^
Free thyroxine (pmol/L^−1^) Ref values: 12.0–22.0	18.4	16.6	16.6

All data presented are values retrieved at baseline (before the intervention) at the beginning of week 18 and following 2 days of energy repletion. Each endocrine marker’s reference values were derived from the assay manufacturer (Roche Diagnostics) validation reports. SHBG, sex hormone-binding globulin; Ref, reference.

* Within the lowest 25% quartile of the reference value.

** Below the reference value.

### 2.5 Strength assessment

Strength was assessed using absolute and relative one repetition maximum (1RM) determination for three multi-joint exercises: back squat, bench press, and deadlift. 1 RM testing was performed at baseline and post-competition at the same time of day and followed a progressive loading format, with an inverse relationship between repetitions and load until the athlete reached failure on their maximal load for one repetition. Considering the athlete’s competitive powerlifting experience, the athlete did not undergo familiarisation with the 1RM test before the intervention. Table 3 shows the baseline and post-competition results (week 19).

**Table 3:** One repetition maximum determination for three multi-joint exercises at baseline and week 19.

Exercises	Baseline (weight 81.8 kg) Absolute 1 RM (1RM relative to BM)	Week 19 (weight: 77.8 kg) Absolute 1RM (1RM relative to BM)	Absolute change (%)	Relative change (%)
Back squat (kg)	200.0 (2.44)	180.0 (2.31)	−20.0 (−10%)	−0.13 (−5.4%)
Bench press (kg)	135.0 (1.65)	125.0 (1.61)	−10.0 (−7.4%)	−0.04 (−2.6%)
Deadlift (kg)	240.0 (2.93)	220.0 (2.83)	−20.0 (−8.3%)	−0.11 (−3.6%)

### 2.6 Psychological assessment

As per a previous case study on a physique athlete (30), the Brunel Mood Scale (BRUMS) was used to assess mood state and completed at baseline and every Saturday morning by the athlete until the competition date (31). Baseline, week 8, week 16, and week 18 results are shown in Figure 1d.

### 2.7 Oral temperature

The athlete was provided with two oral temperature thermometers (Digital Thermometer iProven) and advised to measure his temperature in triplicate immediately upon waking every morning before consuming fluids. Weekly averages are shown in Figure 1b.

### 2.8 Resting heart rate

The athlete tracked his resting heart rate (RHR) using a Samsung Gear 2 Smart Watch immediately upon waking every morning for 18 weeks. Weekly averages are shown in Figure 1b.

### 2.9 Dietary intake

The athlete tracked every item of food and drink consumed using a digital scale and added their data to the app MyFitnessPal. During the first week of the intervention, the accuracy of their dietary report was verified by manually replicating the athlete’s dietary intake into Nutritics (Nutritics Ltd., Dublin, Ireland). The lead author assessed the data every Wednesday and Saturday throughout the intervention period to ensure compliance. Furthermore, the lead author retrospectively cross-referenced the athlete’s dietary intake with more than 50 video blogs recorded by the athlete during contest prep to ensure accuracy.

### 2.10 Intervention

The athlete’s REE (REE_ratio_ 0.99) and endocrine markers (all within physiological reference ranges) at baseline indicated sufficient EA (see Tables 1, 2). In addition, the athlete’s self-reported BM oscillated ≤1% 2 weeks before the start of the intervention; thus, the athlete’s self-reported off-season calorie intake was used as the foundation to program their contest prep diet. At baseline, the athlete was assigned an undulated intake of 2,100 kcal.d^−1^ for five consecutive days and 2,600 kcal.d^−1^ for 2 days (arithmetic mean reported intake 2,276 ± 246 kcal [week 2]). We aimed to sustain a rate of BM loss of ~0.5% per week to maximise muscle retention in accordance with evidence-based recommendations (3). By week nine, the athlete’s dietary intake was adjusted to 2,100 kcal.d^−1^ for six consecutive days, with one higher intake of 2,600 kcal.d^−1^ (arithmetic mean reported intake 2,173 ± 196 kcal [week 9]). By the final month, the athlete’s daily energy intake was reduced by a further 100 kcal (arithmetic mean reported intake 2066 ± 193 kcal [week 17]). During the athlete’s three-day energy repletion period before competition (“peaking”), the athlete was assigned a calorie intake of 2,450 kcal (arithmetic mean reported intake 2,458 ± 4 kcal; see Supplementary Table 2).

The athlete’s protein intake was 2.4 g.kg^−1^.d^−1^, which remained until week 17, when it was reduced to 2.1 g.kg^−1^.d^−1^ to accommodate an increase in carbohydrates during energy repletion (“peaking”). The athlete’s carbohydrate intake was 2.9 g.kg^−1^.d^−1^ until week 18, when it increased to 4.8 g.kg^−1^.d^−1^ for three consecutive days during energy repletion, with an increase in sodium and water on the day of competition to accentuate vascularity and muscle volume (32). The athlete consumed five protein-rich meals (0.25–0.5 g.kg^−1^ per meal) spaced evenly throughout the day to optimize muscle anabolism and protein balance (33). Before training, the athlete consumed a carbohydrate and protein-rich meal (~2 h before) to increase carbohydrate availability and a further 0.5 g.kg^−1^ of protein within 90 min of training finishing to potentiate the post-exercise muscle protein synthetic response (34). Furthermore, the athlete consumed a protein snack of a non-whey source ~2 h before bedtime to increase overnight protein balance (35).

Figure 1e presents the athlete’s weekly training volume. The athlete completed four resistance training sessions weekly, focusing on each muscle group 2–4 times per week, with an average weekly set volume of 71 ± 9 sets and a rep volume of 238 ± 10 reps per week. The athlete incorporated blood flow restriction training at the end of workouts as an alternative training stimulus for the final 4 weeks before the competition. The athlete performed two “350 kcal target” long-intensity steady-state (LISS) cardiovascular sessions per week at baseline. By week 6, the athlete increased their LISS training three times per week; by week 13, this increased to four times per week.

## 3 Statistics

Data are presented as arithmetic means and standard deviations (±). Data was analyzed using Microsoft Excel (version 2021, Microsoft Corporation, WA, USA). Normality was determined using the Shapiro–Wilk test. Pearson’s correlation (normal distribution) and Spearman’s rank correlation (non-normal distribution) were used to test associations for oral temperature, resting heart rate and BM. Statistical significance was set at an alpha of *p* < 0.05. As internal replicate measurements of all endocrine markers were not performed, the inter-assay CoV% provided by Roche Diagnostics for the cobas e 602 analyzer was used as a proxy measure. The typical error (TE) was estimated to determine the variability of each endocrine measure and calculated as the inter-assay CoV% multiplied by the mean measurements by the assay manufacturer divided by 100. The TE multiplied by 2 was used to discern a value indicative of a meaningful change.

## 4 Results

### 4.1 Endocrine, metabolic, psychological, and performance parameters

Between weeks 1–18, the athlete’s free T3 and TT levels fell by −37.2% and −36.8%, falling into clinically low and sub-clinically low ranges, respectively. Follicular stimulating hormone (FSH), LH, and free thyroxine (T4) trended downwards but remained within their reference ranges, whereas sex hormone binding globulin (SHBG) remained largely unchanged. After 2 days of modest energy repletion (2,458 ± 4 kcal.d^−1^, 4.8 g.kg^−1^ BM.d^−1^ of CHO), free T3 increased by 18.5%, returning to within reference range, albeit within the lowest 25% quartile. TT levels decreased by a further −20.9%, reaching clinically low levels in accordance with a − 19.4% decrease in LH (see Table 2 for measured values). The athlete’s REE decreased from baseline by −519 kcal (REE_ratio_ 0.99 to 0.78), and RER decreased from 0.95 to 0.85 by week 18. From week 1 to 17, the athlete averaged a weekly BM loss of −0.30 kg (−0.38% of BM loss per week). The athlete’s sum of eight skinfolds was reduced by −15.7 mm (50.8 mm to 35.1 mm). The athlete’s BRUMs assessment for confusion, depression, and tension remained below average throughout the intervention. Fatigue increased above average at week 8, and vigor and anger were slightly above average during weeks 16–18. The athlete demonstrated decreases in absolute 1 RM in the squat, bench, and deadlift from baseline by −10.0%, −7.4%, and −8.3%, respectively, while their relative 1 RM decreased by −5.4%, −2.6%, and −3.6%.

### 4.2 Physiological associations with reductions in body mass

Considering previous associations between metabolic rate, oral temperature, and energy restriction (36) and reports of a reduction in resting heart rate during contest prep in natural bodybuilders (6, 30), we tested for associations between oral temperature, resting heart rate and BM during the 18-week intervention. As shown in Figure 1b, the athlete’s oral temperature (r = 0.674, *p* = 0.002) and RHR (r = 0.560, *p* = 0.016) tended to decrease with BM throughout the intervention period.

## 5 Discussion

This case study assessed the nature of two primary markers of low EA (free T3 and TT) following 2 days of modest energy repletion. Moreover, we provide further insight into endocrine, physiological, psychological, and strength performance outcomes following a prolonged period of BM loss. The athlete’s free T3 and TT fell into clinically low (2.7 pmol/L^−1^) and sub-clinically low (9.1 nmol/L^−1^) ranges by week 18, respectively. Previous research on male bodybuilders, a combat athlete and military personnel during chronic phases of energy restriction have demonstrated similar reductions (5, 6, 15, 16). Considering the secretion of LH regulates the production of testosterone in males via the anterior pituitary gland secondary to the pulsatile release of gonadotropin-releasing hormone (GnRH) within the hypothalamus, it was surprising that LH, albeit trending downwards, remained largely unchanged between baseline and week 18 compared to TT. Similarly, we anticipated free T4 production to decrease per free T3 and REE and SHBG production to increase per a reduction in TT; however, neither demonstrated meaningful changes. Discrepancies between LH, SHBG and testosterone have been previously documented in male athletes (37, 38) and could have been due to the small number of measurements taken. Moreover, discrepancies in T4 with decreases in T3 have also been reported in the literature (9, 39). However, LH levels from baseline to the athlete’s third measurement (2 days of energy repletion) fell by −23.7%, in conjunction with a −50.0% reduction in TT, suggesting that a reduction in GnRH mediated the decrease in TT.

After 2 days of modest energy repletion, free T3 increased by 18.5%, whereas TT levels continued to decrease by −20.9%. In a recent study of a male combat athlete, a two-day *ad-libitum* increase in calories (64–89 kcal.kg FFM^−1^ BM.d^−1^) led to clinically low testosterone levels increasing more than two-fold, returning close to baseline values in a week (16). Similar increases in free testosterone have been demonstrated in explorers 3 days after completing an 850-km cross-country skiing expedition to the North Pole. While the explorer’s energy repletion intake was not measured in the 3 days following the expedition, a 5 kg weight regain at 5 days indicates a pronounced calorie increase above energy balance (40). The discrepancy between our results and those mentioned is likely due to both studies’ energy repletion intake exceeding total daily energy expenditure requirements and thus reaching optimal EA values to support the restoration of endocrine function. In contrast, if we consider the athlete’s pronounced reduction in REE, our intake likely achieved close to energy balance but below adequate EA to support the restoration of the hypothalamic–pituitary-gonadal axis. Thus, we can speculate that the continued reduction in TT following 2 days of modest energy repletion could suggest that the hypothalamic–pituitary-gonadal axis requires a greater energy and/or carbohydrate intake to respond during periods of short-term energy repletion following energy restriction compared to the hypothalamic–pituitary-thyroid axis; however, due to the study design and limitations described below, this warrants further study.

A reduction in REE appears to be a consistent finding in case studies during contest prep, with reductions in REE ranging from −179 kcal to roughly ~1,000 kcal (5, 6, 20, 30) and a decrease in RER (30). While the use of a predictive equation specific to physique athletes provided a close REE_ratio_ at baseline (0.99) and detected energy deficiency at week 18 (0.78) with the cut-off ratio of <0.90 (mREE/pREE) (27), due to the substantial decrease in the athlete’s REE, common predictive equations used in athletes such as the Harris-Benedict (41) (0.81) and Ten-Haaf (42) (0.75) equations also produced an mREE/pREE ratio of <0.90. From week 1–17, the athlete averaged a weekly BM loss of −0.30 kg, which is lower than the average (−0.42 kg) of other male physique athletes during contest prep (43) but aligns with current evidence-based recommendations (32). Notably, the athlete’s lowest weight was recorded at week 15 (76.3 kg); however, during weeks 16–18, the athlete complained of gastrointestinal (GI) symptoms (bloating and constipation), which likely contributed to the athlete’s lack of BM loss in the final 2 weeks. Upon highlighting these GI complaints, a review of the athlete’s dietary intake was performed to see if any potential dietary culprits of GI distress could be identified (e.g., uncharacteristically high amounts of FODMAP-rich foods) (44), but no abnormal changes from the athlete’s previous 15 weeks of dietary intake had occurred. GI disturbance has been implicated in the REDs physiological model and reported in male athletes with secondary exercise dependence and disordered eating (14, 45); however, further studies are warranted in male athletes. In the final weeks leading up to the competition, the athlete moved house while maintaining his case study, video blogging, and work commitments. In the athlete’s video blog during this time, he mentions stress may have been the cause of his GI disturbance, which is empirically supported (46).

The athlete’s oral temperature decreased throughout the intervention period in accordance with BM losses (r = 0.674, *p* = 0.002; Figure 1b), as per previous research during a period of energy restriction (36). A similar relationship was demonstrated with the athlete’s RHR (r = 0.560, *p* = 0.016), which was reduced by 9 beats.min^−1^ by week 16, akin to previous case reports during contest prep (6, 30). As shown in Figure 1d, the athlete’s mood state remained largely undisturbed during contest prep. However, vigor and anger increased slightly above average in the final 2 weeks. Mood disturbances have been previously reported during contest prep (7), with pronounced increases reported in one physique athlete in the final stage before competition (6). While a heightened preoccupation with food could have been partly causal of the athlete’s mood disturbance, per previous research (7), it may have also been a result of changes in his personal life noted earlier, and the emotional toll on his relationships—both of which were mentioned in the athlete’s video blogs. The athlete demonstrated decreases in absolute 1 RM in the squat, bench, and deadlift from baseline by −10.0%, −7.4%, and −8.3%, while their relative 1 RM decreased by −5.4%, −2.6%, and −3.6%, respectively. Notably, these changes are within the CoV (0.5–12.1%) of a recent systematic review on the retest reliability of the 1RM test (47); thus, some of the reduction in strength could simply be a product of day-to-day variability.

While our study measured indicators of EA, we did not directly measure EA due to discrepancies in skinfold body fat prediction equations (29) and dietary energy intake and exercise energy expenditure both being highly susceptible to error (48, 49). Moreover, it is likely that these measurements alone are not a true reflection of an athlete’s EA, considering non-exercise activity thermogenesis is not factored into the equation and can surpass exercise activity expenditure values in certain athletes (50). Several limitations are worthy of note with this research, which the reader must consider when evaluating the finding’s validity and translatability to practice. The athlete was recruited for the intervention just 2 weeks before its commencement, which prevented test re-test reliability measurements for oral temperature, morning RHR, BRUMS, and 1RM testing. Furthermore, the athlete’s baseline BRUMS assessment was performed on just 1 day, which could have been influenced by transient external factors and the reason for the discrepancy between scores at baseline and during the intervention. Internal quality control metrics of the endocrine measurements were not performed; thus, the inter-assay CoV was derived from the assay manufacturer as a proxy measure. The Metallyzer 3B (Cortex) system used to assess RER at rest in our study has been shown to overestimate carbohydrate oxidation by 53% and underestimate the energy derived from fat by 25%, albeit in an exercise context (51). While our results indicate a shift towards greater fat oxidation similar to previous research on a physique athlete during contest prep (30), caution is advised when interpreting the RER values due to the potential for measurement inaccuracies. Furthermore, the athlete’s free T3 levels were assessed using the immunoassay method, which, compared to the gold-standard method of liquid chromatography–tandem mass spectrometry, frequently overestimates free T3 levels at the low reference interval (52). Lastly, the athlete’s TT levels were measured at three *single* time points, when it is recommended to take two consecutive measures at each time point due to day-to-day variation (53).

## 6 Practical perspective

Our case study offers insight into the nature of two primary indicators of low EA in males (free T3 and TT) as per the IOC REDs CAT2 during chronic energy restriction and a short-term period of modest energy repletion. Moreover, this study offers further insight into the effects of “potential indicators” of low EA (e.g., REE) during 18 weeks of contest prep (13). The athlete adopted evidence-based weight-loss recommendations for athletes yet incurred declines in metabolic and reproductive hormones, resulting in a risk status of “Moderate to High” (Orange) according to the IOC REDs CAT2 Severity/Risk Assessment Tool (13, 14). The extent to which these alterations resulted from the athlete’s low EA load or degree of leanness was not determined and requires further insight; however, the results are pertinent to practitioners working with athletes adopting similar weight-loss approaches. The implications of the transient nature of free T3 and the unresponsiveness of TT following a 2 days modest energy repletion warrant further study and could have a meaningful impact on assessing low EA in males. We successfully documented endocrine, physiological, psychological, and performance indicators while the athlete adopted evidence-based guidelines for contest prep. Importantly, our case study was undertaken using assessment methods accessible to many research-active practitioners, which we hope will allow more practitioners to report their inductions from practice to inform future research, bridging the gap between science and practice (54).

## 7 Case perspective

The athlete felt he achieved his best contest prep condition and won his physique category. Reflecting on the intervention the day before the competition, the athlete questioned whether 18 weeks of energy restriction, necessitating constraints on social events, travel, and causing a strain on his close relationships, coupled with an ongoing heightened fixation with food, was worth the reported changes in body composition. Several days after the show, the athlete mentioned that his motivation, as a result of no longer working towards the competition, had noticeably reduced. A week post-contest prep, the athlete increased his weight by ~1.5 kg but was not preoccupied with the rise in BM, unlike previous post-contest prep experiences. He credited this change in perspective to viewing his increased BM as an indicator of returning to a condition commensurate with achieving his best lifting performances.

## Data Availability

The original contributions presented in the study are included in the article/Supplementary material, further inquiries can be directed to the corresponding author.
