# Supplementary material for: From semi-starvation to the stage: a case report on indicators of low energy availability in a drug-free bodybuilder during contest preparation and peak week
Source: Front Nutr. 2024 Nov 13;11:1465001. doi: 10.3389/fnut.2024.1465001 (PMC11601077; doi:10.3389/fnut.2024.1465001)
Supplement: Supplementary file 1 [file Table_1.docx]

**Supplementary Table 1.** **Three-time point mean during the intervention, mean measurements by assay manufacturer, inter-assay CV%, sensitivity, typical error and typical error multiplied by 2.**

| **Endocrine markers** | **Three-time point mean** | **Mean measurements by assay manufacturer** | **Inter-assay  CoV%** | **Sensitivity** | **Typical error** | **2TE** |
| --- | --- | --- | --- | --- | --- | --- |
| Follicular stimulating hormone (IU/L^-1^)  Ref values: 1.5 - 12.4 | 6.1 | 5.33 | 3.60 | 0.10 | 0.192 | 0.384 |
| Luteinizing hormone (IU/L^-1^)  Ref values: 1.7 - 8.6 | 3.4 | 5.81 | 2.0 | 0.10 | 0.116 | 0.232 |
| DHEA sulphate (umol/L^-1^)  Ref values: 4.34 - 12.2 | 9.8 | 10.70 | 2.7 | 0.003 | 0.289 | 0.578 |
| Total testosterone (nmol/L^-1^)  Ref values: 8.6 - 29.0  **Lowest 25% quartile of the ref values:**  8.6 - 13.7 nmol/L | 10.2 | 7.39 | 3.2 | 0.09 | 0.236 | 0.473 |
| SHBG (nmol/L^-1^)  Ref values: 18.3 - 54.1 | 27.0 | 13.7 | 1.8 | 0.35 | 0.247 | 0.493 |
| Cortisol (nmol/L^-1^)  Ref values: 166 - 507 Morning 6-10 am | 461.7 | 551.0 | 2.3 | 1.50 | 12.673 | 25.346 |
| Free triiodothyronine (pmol/L^-1^)  Ref values: 3.1 - 6.8  **Lowest 25% quartile of the ref values:**  3.1 - 4.0 pmol/L | 3.4 | 3.5 | 3.2 | 0.60 | 0.112 | 0.223 |
| Free thyroxine (pmol/L^-1^)  Ref values: 12.0 - 22.0 | 17.2 | 17.5 | 2.6 | 0.30 | 0.455 | 0.910 |
| ***Note:*** The three-time point mean is the cumulative average of each endocrine marker during the intervention (baseline, week 18, and two days of energy repletion). The mean measurements by the assay manufacturer (Roche Diagnostics) reflect multiple assay runs in duplication on consecutive days. For instance, the mean measurement by Roche Diagnostics for free T3 reflects 84 samples (two runs per day in duplication for 21 days). The inter-assay coefficient of variation (CoV) and sensitivity (the lowest analyte concentration that can be detected) for all endocrine markers were sourced from Roche Diagnostics validation report for the cobas e 602 analyzer. We chose the closest mean value by Roche Diagnostics to the three time-point mean for each endocrine marker during our intervention. The typical error was estimated to determine the variability of each endocrine measure and calculated as the inter-assay CoV% multiplied by the mean measurements by the assay manufacturer divided by 100. The typical error multiplied by 2 was used to discern a value indicative of a meaningful change. TE, typical error. | | | | | | |

**Supplementary 2: Documented calories and macronutrients at week 2, week 9, week 17, and during peaking**

|  | Week 2 | Week 9 | Week 17 | Peaking phase |
| --- | --- | --- | --- | --- |
| Energy (kcal) | 2276 ± 246 | 2173 ± 196 | 2066 ± 193 | 2458 ± 4 |
| Protein (grams) | 190 ± 3 | 190 ± 4 | 185 ± 6 | 160 ± 0 |
| Protein g.kg^-1^ BM.d^-1^ | 2.3 ± 0.0 | 2.4 ± 0.0 | 2.4 ± 0.1 | 2.1 ± 0.0 |
| CHO (grams) | 242 ± 59 | 224 ± 42 | 233 ± 54 | 370 ± 7 |
| CHO g.kg^-1^ BM.d^-1^ | 3.0 ± 0.7 | 2.8 ± 0.5 | 3.0 ± 0.7 | 4.8 ± 0.1 |
| Fat (grams) | 58 ± 4 | 58 ± 4 | 44 ± 2 | 38 ± 4 |
| Fat g.kg^-1^ BM.d^-1^ | 0.7 ± 0.0 | 0.7 ± 0.0 | 0.6 ± 0.0 | 0.5 ± 0.0 |
| ***Note:*** All data presented are mean values taken at Week 2 of the intervention, Week 9 of the intervention, Week 17 and during the  athlete's energy repletion phase (‘peaking’). BM = body mass, CHO = carbohydrates, pw = per week. Week 2 data was chosen to illustrate  the athlete’s initial dietary intake as the athlete started tracking their intake on Tuesday of Week 1, preventing a seven-day average. | | | | |
